# What Is the Impact of Diet on Nutritional Diarrhea Associated with Gut Microbiota in Weaning Piglets: A System Review

**DOI:** 10.1155/2019/6916189

**Published:** 2019-12-26

**Authors:** Jing Gao, Jie Yin, Kang Xu, Tiejun Li, Yulong Yin

**Affiliations:** ^1^National Engineering Laboratory for Pollution Control and Waste Utilization in Livestock and Poultry Production, Institute of Subtropical Agriculture, The Chinese Academy of Sciences, Changsha, China; ^2^Key Laboratory of Agro-Ecology, Institute of Subtropical Agriculture, The Chinese Academy of Sciences, Changsha, China; ^3^University of Chinese Academy of Sciences, Beijing, China; ^4^College of Life Science, Hunan Normal University, Changsha, Hunan, China

## Abstract

Piglets experience severe growth challenges and diarrhea after weaning due to nutritional, social, psychological, environmental, and physiological changes. Among these changes, the nutritional factor plays a key role in postweaning health. Dietary protein, fibre, starch, and electrolyte levels are highly associated with postweaning nutrition diarrhea (PWND). In this review, we mainly discuss the high protein, fibre, resistant starch, and electrolyte imbalance in diets that induce PWND, with a focus on potential mechanisms in weaned piglets.

## 1. Introduction

Weaning is sudden and stressful and one of the most challenging periods in a pig's life [1]. Newly weaned pigs are usually stressed by nutritional, psychological, environmental, physiological, and social factors [2, 3]. Because of such stressors, piglets are often characterized with reduced growth performance and an increased prevalence of diarrhea after weaning [4, 5]. When undergoing the transition from a milk-based diet to a weaned diet, the piglets suffer a severe decrease in feed intake for a couple of days after weaning [6]. Furthermore, in order to adapt to the new environment, the composition of the gastrointestinal microbiota is also modified as a result of changes in feeding behavior and diet composition [6]. This period is often associated with a growth challenge because of a high incidence of gastrointestinal disorders, such as PWND [7].

Postweaning diarrhea is considered a major health problem and causes substantial morbidity and mortality in livestock [8, 9]. It is well established that postweaning diarrhea is a multifactorial gastrointestinal disease, and undernutrition has major etiological factors [10–12]. The gastrointestinal tract is a complex, balanced ecosystem [4, 13]. The dietary composition is a major factor influencing the intestinal microbial ecosystem [14, 15]. Hence, considering the balance between the intestinal microbial ecosystem and the composition of the diet, postweaning nutritional diarrhea (PWND) is a major problem during the postweaning period [11, 16].

The most efficient manner to alleviate the degree of PWND is to regulate the nutritional composition of the diet [15, 17]. Various nutritional approaches for improving the weaning transition and alleviating enteric diseases have been researched over the past several years [11, 18]. Evidence suggests that specific dietary interventions, such as the control of protein [19, 20], fibre [21], starch [22], electrolyte balance [23], and other constituents in the daily diet, could reduce the proliferation of certain PWND [11, 24, 25]. The purpose of the present review is to summarize several common kinds of PWND in order to better expound the role of nutrition in causing and modulating PWND in pigs.

## 2. High Protein Level Induces PWND

To decrease PWND, piglets are usually given antibiotics; however, antibiotics have been banned for use in livestock for human consumption. Thus, researchers have focused on finding a replacement for antibiotics in piglet feeding. One of the alternatives is the feeding of low-protein diets [26–28].

### 2.1. The Effect of Dietary Protein on Growth Performance and Digestibility of Nutrients of Weaned Piglets

The consumption of a low crude protein (CP) diet, which has direct effects on PWND, reduces the availability of substrates for bacterial fermentation and improves fecal consistency [29–31]. Dietary CP and individual AAs (amino acids) both affect the formation of metabolites during microbial fermentation [32]. However, high dietary CP concentration for early-weaned piglets could increase microbial fermentation of undigested protein and increase the proliferation of pathogenic bacteria in the gastrointestinal tract [3]. An excessive supply of dietary protein induces protein fermentation by intestinal microbiota in piglets [33]. Volatile fatty acids (VFAs) and potentially toxic compounds produced by bacterial fermentation of undigested protein substances, such as ammonia and amines, can reduce the growth performance of piglets [34, 35]. The increased production of amines has been found to increase the incidence of diarrhea at weaning in pigs [36, 37].

### 2.2. The Effect of Dietary Protein on the Gut Health of Weaned Piglets

It is well known that both exogenous and endogenous source proteins can be used by the gastrointestinal microbiota as a fermentable substrate [38, 39] and can be used for the production of diet proteins through degradation, including branched-chain fatty acids (BCFAs), ammonia, amines, phenols, and indoles [35, 39]. Bacteria, such as *Bacteroides* spp., *Propionibacterium* spp., *Streptococcus*, and *Clostridium* species, are associated with the formation of the substances listed above [20]. For example, BCFAs are produced by *Clostridia* [40]. Furthermore, intestinal concentrations of BCFAs possibly are used as indicators for the extent of protein fermentation [41].

Protein fermentation results in the production of metabolites that are in direct contact with the colonic mucosa and can directly interact with the mucosal cells. Undigested dietary protein and proteins of endogenous origin transfer to the large intestine for fermentation to toxic metabolites, such as ammonia, biogenic amines, and hydrogen sulfide. Most of these products can impair epithelial integrity and promote inflammatory reactions [34, 42]. Then, the metabolites or bacterial toxins may reduce the ability for fluid reabsorption and mask small intestinal hypersecretion [29]. An increased concentration of ammonia was found in parts of the intestinal tract of piglets fed high-protein diets [3]. Infusion of ammonium chloride from the isolated distal colon increased the proliferation of epithelial cells in rats, which may contribute to the development of gastrointestinal disorders [43]. Biogenic amine concentrations increased in the hindgut when feeding on highly fermentable protein, and bacterial putrescine played a role in the potential detrimental effects on gut health by decreasing the energy supply to the colonocytes [44]. Hydrogen sulfide impacts gut health by breaking down the mucus layer and by increasing the permeability of the mucus barrier [45]. According to several studies, high protein fermentation is associated with an increased risk of cancer [46].

### 2.3. The Effect of Dietary Protein on the Incidence of PWND

PWND is a gut disease induced by the stress of nutrition and is characterized by an increase in the microbial fermentation of supernumerary proteins [47]. Additionally, watery feces, decreased growth performance, high morbidity, and mortality have been noticed to occur with PWND [48]. However, the mechanism between protein fermentation and the gastrointestinal tract (GIT) is still unknown. Some studies showed that a high-protein diet led to a higher incidence of PWND [6, 35]. Interestingly, an increase in ammonia concentration has a detrimental effect on the health of the GIT and a negative effect on the growth and differentiation of intestinal epithelial cells [49, 50]. Additionally, BCFAs and ammonia are toxic metabolites for the intestinal mucosa and most likely trigger PWND and the poor performance in piglets [39, 51, 52]. More importantly, the upregulated expression of ammonia may induce a disorder of the intestinal microbial balance during weaning [20]. Additionally, the initially predominant lactobacilli decrease in number during weaning, leading to the downregulation of the GIT immunity and the formation of short chain fatty acids (SCFAs) [53]. More importantly, piglets fed a high-protein diet experience a high buffering capacity [54], an increase in the small intestinal pH [26], and a decrease in the expression of SCFAs, mainly butyrate, which probably permit a quick recovery of the intestinal epithelium, reducing the incidence and severity of PWND [55].

**Figure 1:**
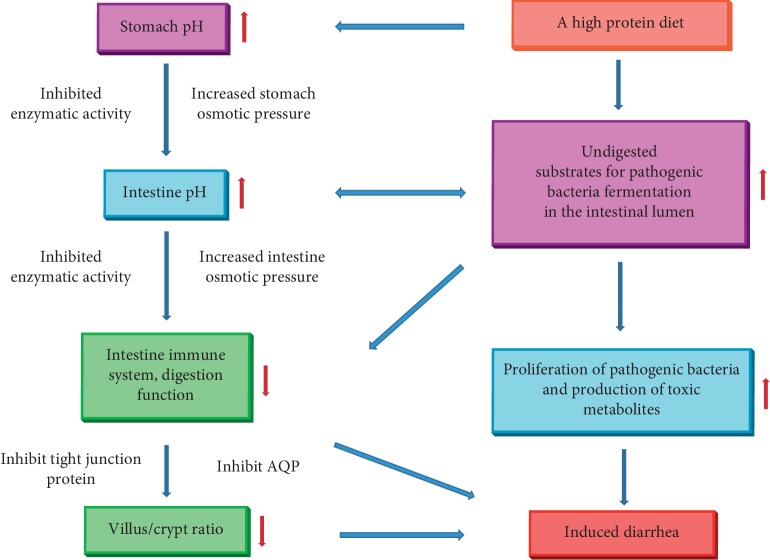
The possible mechanism of high protein diets induced postweaning nutritional diarrhea. AQP: aquaporin.

In summary, a high-protein diet increases the expression of BCFAs and ammonia, which can promote the growth of pathogenic bacteria, while a low-protein diet promises an increase in the expression of SCFAs, which may result in the establishment of beneficial microbes [56, 57]. Therefore, with a low-protein diet, beneficial bacteria rapidly proliferate and occupy the binding sites on the intestinal mucosa that could otherwise be occupied by pathogenic bacteria [58]. These differences between protein levels may reduce the incidence and severity of PWND and improve the growth performance of piglets [3]. Thus, it can be concluded that choosing a low-protein diet to feed postweaned piglets may be an effective way to decrease PWND incidence [3] (Figure 1).

## 3. The Effect of Dietary Fibre on PWND

Fermentable carbohydrates constitute the major energy source for microbial fermentation and therefore may act as a link between the piglet and its enteric commensal microbiota [59, 60]. Furthermore, dietary fibre may be beneficial for gut health and decreases diarrhea incidence in pigs [61, 62]. And significant effect on diarrhea incidence was observed in the pigs fed the fibre source diet compared with the pigs fed the control diet [63, 64]. A difference in diarrhea incidence was observed among the different sources of fibre diets [60].

### 3.1. The Effect of Dietary Fibre on Growth Performance and Digestibility of Nutrients of Weaned Piglets

Adding fibre in the daily diet could improve the adaptation of the pigs during the weaning period [64]. Depending on the kinds of fibrous ingredients, the effects of feeding high-fibre diets on the performance of the piglets differed [62, 63]. The impact of dietary fibre on piglets' nutrition might be determined by the properties of fibre and/or fibre sources [65, 66]. For example, the fibre in the wheat bran diet was adapted by piglets and acted as prebiotics [60]. Wheat bran is a kind of insoluble fibre and when added to the weaned piglets' diet, it appears that it is related to a higher feed intake and development of the gastrointestinal tract [67, 68]. More research is necessary to clarify the effects of the dietary fibre composition on the growth performance of weaned piglets.

### 3.2. The Effect of Dietary Fibre on the Gut Health of Weaned Piglets

In consideration of intestinal bacteria, fibre diets influenced the health of piglets around the time of weaning [69, 70]. Previous studies showed that a lower villus height: crypt depth ratio is associated with microbial challenges and antigenic components of the feed [71, 72]. Moreover, a study of intestinal mucosal morphology was used to evaluate the surface area of the intestine undertaken for mucosal integrity [73, 74]. Adding wheat bran fibre to the daily feed elevated the ileal mucosal integrity by improving the ileum villus height and the villus height: crypt depth ratio, which is in agreement with previous findings that showed that feeding high-insoluble-fibre diets protected against pathogenic bacteria by increasing the villus length [75, 76]. Furthermore, research has shown that piglets fed soluble and insoluble dietary fibre had more goblet cells in the ileum than did fibre-free piglets [77, 78]. The goblet cells played an important role in the intestine by synthesizing and secreting several mediators, mainly found in the small and large intestine, that were resistant to proteolytic digestion and stimulated the repair process, such as mucin and peptide trefoil factors [24, 79]. Studies suggested that piglets fed a fibre diet had a higher TGF-*α* concentration in their colons than that of other fibre-free groups [80]. Altogether, a fibre diet could improve the intestinal barrier function by increasing the concentration of factors associated with intestinal barrier function. However, different dietary fibre compositions induce different changes in the intestinal bacteria [81, 82].

### 3.3. The Effect of Dietary Fibre on the Incidence of PWND

Dietary fibre has been reported to improve gut health and decrease the diarrhea incidence in pigs [61, 83]. A wheat bran diet has been shown to decrease the amount of pathogenic *E. coli* in the feces and reduce the incidence of PWND [61, 84]. It is reported that a pea fibre diet could improve the intestinal health in animals by reducing the adhesion and increasing the excretion of enterotoxigenic *E. coli*, and such a diet could reduce the incidence of PWND as well [85]. However, the effect of a fibre diet on the incidence of diarrhea was not observed between the piglets fed fibre diets and the control group. However, a difference in diarrhea incidence was observed among the fibre source diets [64, 86]. The mechanism of the effects of the dietary fibre source on the incidence of diarrhea in weaned piglets may result from the inconsistent intestinal function in regulating intestinal bacteria [87]. Previous studies have shown that feeding weaned piglets high-insoluble-fibre diets might better protect them against pathogenic bacteria by increasing the villus length [85, 88]. The intestinal barrier integrity reflects the paracellular space between epithelial cells and may prevent the paracellular diffusion of intestinal bacteria across the epithelium [89]. Additionally, tight junction proteins play a key role in intestinal barrier integrity [90]. The probiotic *Lactobacillus* can increase occludin gene mRNA levels of Caco-2 cells, and *E. coli* decreases the levels of ZO-1, occludin, and claudin-1 tight junction complex in the epithelial cells, proving that bacteria affect the integrity of the intestinal barrier by regulating the gene expression level of tight junction proteins [91, 92]. The effect of fibre on tight junction proteins is associated with the number of bifidobacteria and lactobacilli [93]. Fibre in the diets has also promoted intestinal proinflammatory cytokine (IL-1 and TNF-*α*) mRNA levels as interference factors of the intestinal barrier [94, 95]. However, more research between PWND and intestinal bacteria alterations mediated by fibre in the diet must be done.

**Figure 2:**
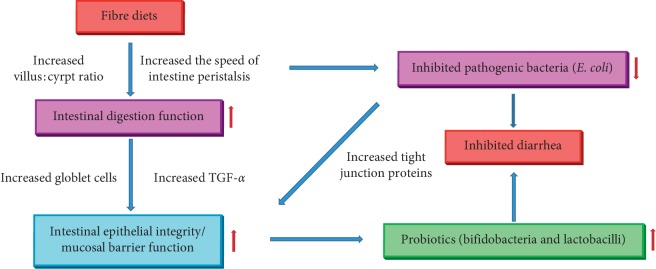
The possible mechanism of fibre diets regulated postweaning nutritional diarrhea. TGF-*α*: transforming growth factor-*α*.

Complex fibre sources could affect the intestinal mucosal barrier function and regulate intestinal bacteria in weaning piglets. Additionally, fibre composition is considered to be an important factor affecting the intestinal barrier function in piglets and could induce the incidence of PWND (Figure 2).

## 4. The Effect of Dietary Electrolyte Balance on PWND

### 4.1. Dietary Electrolyte Absorption in the Intestine

The intestinal lumen accepts 8–10 L/day of fluid, containing ingested food and biological secretions. The small intestine absorbs the highest percentage of this fluid content, and the last 1.5–1.9 L of the fluid is absorbed by the large intestine [96]. Otherwise, <0.1–0.2 L/d of the fluid content is excreted in the feces in an abnormal condition [97]. However, piglets around the time of weaning suffer a significant reduction in the colon absorptive capacity, leading to diarrhea [98, 99].

Electroneutral sodium chloride in the intestine is absorbed by luminal Na^+^/H^+^ and Cl^–^/HCO^–^ exchangers [100, 101]. The remaining absorption of sodium chloride is due to transcellular or paracellular absorption of Cl^–^ [102]. Na^+^/H^+^ and Cl^–^/HCO^–^ exchangers in luminal brush-border membranes of the colonic epithelial cells are required to absorb sodium chloride [103]. This process is driven by the action of the Na^+^-K^+^-ATPase and is regulated by Na^+^ depletion [104, 105].

The Na^+^/H^+^ exchangers play a key role in Na^+^ and water absorption and the maintenance of intracellular pH and cell volume [106]. Eight types of Na^+^/H^+^ exchangers named NHE have been defined in the intestinal epithelium. In the intestine, NHE1 (SLC9A1), NHE2 (SLC9A2), NHE3 (SLC9A3), and NHE8 (SLC9A8) have been shown to be present in the intestinal epithelium [105, 107]. NHE1 (SLC9A1) is expressed in the basolateral membrane of the intestinal epithelial cells, is not affected by Na^+^ depletion, and does not contribute to luminal ion and water absorption [108, 109]. NHE2 (SLC9A2) and NHE3 (SLC9A3) are both expressed in the intestinal epithelium, with a larger contribution of NHE3 (SLC9A3) to Na^+^ absorption under control conditions [110]. Otherwise, NHE3 (SLC9A3) is reported as the main transporter for Na^+^ absorption in the intestine [97, 111]. NHE3 (SLC9A3)-knockout mice had reduced intestinal Na^+^ and water absorption and induced diarrhea [112, 113]. Na^+^/H^+^ exchange occurs in both surface and crypt epithelium and might be affected by CFTR Cl^–^ channels [114, 115]. However, the exact impact of CFTR Cl^–^ channels on the regulation of Na^+^/H^+^ exchange in the small intestine is not clear.

In mammalian intestinal epithelial cells, two types of SLC26 gene families, named DRA (SLC26A3) and PAT-1 (putative anion transporter, SLC26A6), have been identified as representing apical Cl^–^/HCO_3_^–^ exchangers [116, 117]. DRA is predominantly expressed in the colon and duodenum, whereas PAT-1 is mainly expressed in the jejunum and ileum [118, 119]. DRA mutations have been found to induce severe diarrhea, massive loss of Cl^–^ in stools, and metabolic alkalosis as well as serum electrolyte imbalance [120, 121]. PAT-1-knockout mice did not present this diarrhea phenotype [122]. In addition, similar to NHE3, a Cl^–^/HCO_3_^–^ exchange is also controlled by CFTR in the colonic epithelium [123, 124]. Taken together, the current studies indicate a regulation of both Na^+^/H^+^ and Cl^–^/HCO_3_^–^ exchangers by CFTR, which therefore play an important role in the electroneutral absorption of sodium chloride and regulation of cellular and mucosal pH in the animal gastrointestinal tract [117, 125, 126].

### 4.2. The Effect of Dietary Electrolyte Balance on the Growth Performance of Weaned Piglets

The animal industry is always concerned about minerals in feed, such as calcium (limestone), phosphorus (calcium phosphate), and sodium and chloride (salt and sodium bicarbonate) [127, 128]. Animal feed adds minerals not only to satisfy the mineral requirements but also to modify the dietary electrolyte balance (EB) [129]. The balance between cation (Na+) and anions (K^+^/Cl^–^) and the acid or alkaline load from the diet may strongly alter the acid-base status and growth performance of weaned piglets [130, 131]. It is reported that an excess of chloride ions induces a negative dietary EB and reduces the growth performance of weaned piglets [132–134]. In short, a dietary addition of minerals in postweaning diets, such as calcium chloride and sodium bicarbonate, could affect the EB and significantly alter the feeding behavior, apparent digestibility, and productive performance of postweaned piglets. Additionally, piglets showed a bias toward low-EB diets, which optimized their performance more so than that for high-EB diets [130, 135].

### 4.3. The Effect of Dietary Electrolyte Balance on the Incidence of PWND

Enteric pathogens have been proven to stimulate intestinal secretion of electrolytes and water [136, 137]. In most species, dietary electrolyte balance may be expressed as Na^+^/H^+^ and Cl^–^/HCO_3_^–^ and has been influenced by proportions of monovalent mineral cations and anions [138, 139]. Electrolyte balance plays a critical role in intestinal phenotype and function [140]. The disorder of daily electrolyte balance after weaning makes a large contribution to postweaning diarrhea, induces severe intestinal electrolyte turbulence, and negatively affects the piglets' growth performance after weaning through excessive loss of salt and water [130, 133]. The gastrointestinal tract exhibits segmental heterogeneity in the various ion transporters and channels in the postweaning period, which play a control role in conjunction and determine the electrolyte content and fluid volume in the lumen. In basic situation, the reason of PWND is the imbalance between the absorption and secretion of ions and solutes across the gut epithelium [141]. This imbalance of electrolytes in the digestive tract of piglets is induced by the presence of bacteria that could deliver toxins into the gut and disturb the development of the epithelium [142]. The enteric pathogens spread rapidly and cause infection in the piglets' intestines [143]. This situation results in the formation of watery feces, or PWND, in combination with reduced growth performance, morbidity, and even mortality of postweaning piglets.

**Figure 3:**
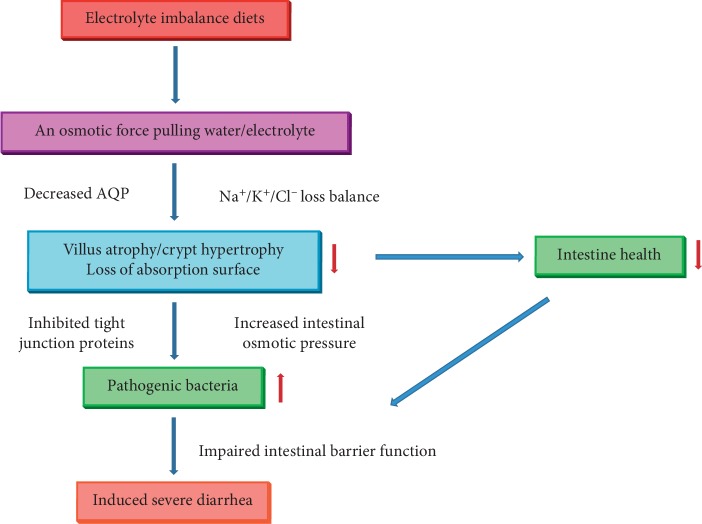
The possible mechanism of electrolyte imbalanced diets induced postweaning nutritional diarrhea. AQP: aquaporin.

In principle, the processes that result in induced PWND are proposed as follows. First, postweaning diets contain unabsorbed solutes that exert an osmotic force pulling water and electrolytes into the intestinal lumen [144, 145]. Second, the syndromes result in villus atrophy and crypt hypertrophy, thereby adversely altering the balance of absorption and secretion [146, 147]. Lastly, active secretion is stimulated by unabsorbed dihydroxy bile acid and fatty acid [148]. The altered bile acid and fatty acid transport themselves into the lipid phase of the plasma membrane [149, 150]. Then, the excess fecal water from the decreased intestinal absorption and the increased intestinal secretion is the reason for diarrhea [151, 152]. Generally, the electrolyte imbalance in postweaning diets exhibited alterations in motility, changes in paracellular permeability, loss of absorption surface, a change in electrolyte fluxes in postweaning piglets and, finally, induced PWND [153–155] (Figure 3).

## 5. The Effect of Dietary Starch Content on PWND

Starch is the main carbohydrate source of animal diets and the main energy source required for both animals and humans [156, 157]. It is composed of two types of a-glucan polymers: amylose and amylopectin [158, 159]. Starch has been proven to have a significant effect on the composition and activity of the intestinal microflora, through an improvement of the growth of beneficial bacteria and a reduction in the development of pathogenic bacteria in the intestines [160, 161]. Based on the digestible capacity, starches could be classified into rapidly digestible starch (RDS), slowly digestible starch (SDS), and resistant starch (RS) [162, 163]. RS cannot be absorbed in the small intestine, but it passes to the large bowel and beneficially modifies the gut microbial populations [164, 165].

### 5.1. The Effect of Resistant Starch Source on the Intestine Digestive Ability and Function of Postweaning Piglets

To ameliorate PWND and improve the gastrointestinal function of piglets, one useful alternative is to use a dietary prebiotic material, such as RS [166, 167]. The properties of prebiotics such as RS can act as indigestible carbohydrate substrates for cecal and colonic microbiota that influence the host gut health, as shown in animal and human studies [161, 166]. Starch digestion begins in the mouth. The enzyme *α*-amylase begins to digest starch to oligosaccharides and maltose. Starch does not digest in the stomach but is transported to the small intestine and broken down to glucose and maltose by pancreatic amylase [161, 168]. Then, the small intestine absorbs glucose by active transport, with the residual part being passively diffused through the villi [169, 170]. Starch digestion is influenced by many factors, such as the presence of lipids, proteins, and minerals, the amylose to amylopectin ratio, and digestion conditions [171, 172]. RS starts to be fermented in the large bowel by colonic microflora and then is digested into hydrogen, methane, and short-chain fatty acids (SCFAs), such as acetic, propionic, and butyric acid [173]. In addition, it is said that the concentration of SCFAs is significantly increased in the large intestine after RS consumption [174, 175]. SCFAs play a positive role in colonic muscles and the absorption of calcium, magnesium, and water, and they also positively stimulate the colonic microflora [176, 177]. According to one experiment, a lower level of RS in the diet will decrease the growth challenge seen in postweaning piglets [178]. It is likely that piglets fed diets containing a higher level of RS (14%) exhibited more undigested starch in the ileum compared with that for piglets fed a diet without added RS [179]. It is reported that RS could be hydrolyzed by several Bifidobacterium strains, such as *B. adolescentis*, *B. bifidum*, *B. breve*, *B. infantis*, *B. lactis*, and *B. longum* [180]. In short, the beneficial effects of RS in the large bowel appear mainly because of the appearance of SCFAs formed by the previously mentioned bacterial fermentation [181]. Colonic bacteria ferment RS to SCFAs, mainly acetate, propionate, and butyrate, and benefit the large bowel of the postweaning piglet [166, 182].

### 5.2. The Effect of Resistant Starch Source on the Incidence of PWND

According to the existing research, a diet containing 7% resistant potato starch reduced the PWND incidence compared to that with a diet containing 14% resistant potato starch [178]. In addition, a diet containing 0.5 or 1.0% of resistant potato starch reduced the incidence of PWND and improved the growth performance in weaning piglets [169]. As we mentioned before, the concentration of SCFAs significantly increases in the large intestine after RS consumption [183]. In contrast, the molar proportion of BCFAs decreases. All of these results occurred because of the greater amounts of substrates available for carbohydrate-utilizing microbiota in the colons of pigs fed diets containing RS [184]. BCFAs are a harmful fermentation product and are a predisposing factor for PWND as well [185]. In addition, RS diets in piglets have a sharp reduction in ileal and cecal digesta pH [186]. According to what we know, bacteria ferment organic matter in the large intestine, including RS to SCFAs, and therefore reduce the pH in the GIT. There is a positive relationship between dietary intake of resistant starch and fecal output [187, 188]. Simply put, postweaning piglets fed a diet that includes a low level of RS leads to increases in the concentration of SCFAs, such as acetate, propionate, and butyrate, as well as the concentration of other terminal products, such as lactate, ethanol, succinate, carbon dioxide, hydrogen, and methane [165, 189]. The increasing SCFA levels decrease the gut pH; help raise gut motility; improve the absorption of nutrients, such as calcium, magnesium, and iron; and provide energy for the colonic epithelium and the host [190, 191]. Importantly, low-level RS diets decrease the concentrations of BCFAs, which are a harmful fermentation product as well as a predisposing factor for PWND [165, 192].

**Figure 4:**
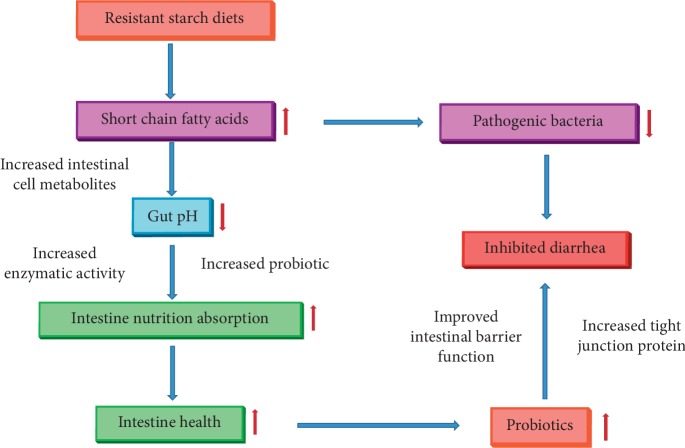
The possible mechanism of resistance starch diets induced postweaning nutritional diarrhea.

We learned that diets containing increased colonic fermentation associated with substrates such as RS also have striking effects on the composition of the gut microflora in order to increase the bacteria populations that are helpful to the bowel and decrease the bacteria populations that are harmful for a healthy large intestine [193, 194]. According to a previous study, lactobacilli and bifidobacteria are considered beneficial and were found to increase in abundance in the cecum with RS diets [195]. Furthermore, a trial that used a diet with lactobacilli strains added decreased the duration and incidence of diarrhea [196]. In short, supplementing weaned pigs' diets with at least 0.5% RS increased the populations of bacteria that are potentially good for the large intestines, such as lactobacilli and bifidobacteria [197], which in turn decreased the amount of bacteria that are harmful for a healthy bowel. Finally, the increase in good bacteria populations and the decrease in harmful bacteria populations resulted in a decreased incidence of diarrhea [198, 199] (Figure 4).

## 6. Conclusion

Weaning is a grand challenge in the swine industry, which frequently induces severe intestinal disorders and gut diseases, raising serious economic and public health concerns. In addition, the gut microbiota derangement induced by changes in the diet of piglets around the time of weaning is the most direct reason of PWND. Despite the progress in modern pig farms during the last decade to prevent infectious diseases and improve global animal health, PWND is still an event that causes significant economic losses in the pig industry. However, we have now learned that the key component that leads to PWND is the composition of the daily diets for postweaning piglets. As described herein, the percentage of protein, fibre, and RS in the diet as well as the electrolyte balance could influence the fermentation products, thus altering the gastrointestinal microbiota composition and, as a result, inducing the incidence of PWND. Clearly, we require well-controlled studies to better understand the impact of nutrition on the growth of piglets around weaning. Controlling the nutrition in the diets is the most promising strategy for the prevention of PWND. The interaction of nutrition along the intestinal tract and the influence it has on the host still must be defined further in order to formulate appropriate “healthy” pig diets.
